# Discovery of Novel IDH1 Inhibitor Through Comparative Structure-Based Virtual Screening

**DOI:** 10.3389/fphar.2020.579768

**Published:** 2020-11-11

**Authors:** Yuwei Wang, Shuai Tang, Huanling Lai, Ruyi Jin, Xu Long, Na Li, Yuping Tang, Hui Guo, Xiaojun Yao, Elaine Lai-Han Leung

**Affiliations:** ^1^ College of Pharmacy, Shaanxi University of Chinese Medicine, Xi'an, China; ^2^ State Key Laboratory of Quality Research in Chinese Medicine, Macau University of Science and Technology, Macau, China; ^3^ State Key Laboratory of Drug Research, Shanghai Institute of Materia Medica, Chinese Academic of Sciences, Shanghai, China

**Keywords:** IDH1, gliomas, molecular docking, virtual screening, docking-based virtual screening

## Abstract

IDH1 mutations occur in about 20–30% of gliomas and are a promising target for the treatment of cancer. In the present study, the performance of aIDH1^R132H^ was verified *via* glide-docking-based virtual screening. On the basis of the two crystal structures (5TQH and 6B0Z) with the best discriminating ability to identify IDH1^R132H^ inhibitors from a decoy set, a docking-based virtual screening strategy was employed for identifying new IDH1^R132H^ inhibitors. In the end, 57 structurally diverse compounds were reserved and evaluated through experimental tests, and 10 of them showed substantial activity in targeting IDH1^R132H^ (IC_50_ < 50 μM). Molecular docking technology showed that L806-0255, V015-1671, and AQ-714/41674992 could bind to the binding pocket composed of hydrophobic residues. These findings indicate that L806-0255, V015-1671, and AQ-714/41674992 have the potential as lead compounds for the treatment of IDH1-mutated gliomas through further optimization.

## Introduction

Isocitrate dehydrogenase 1 (IDH1) is a critical metabolic enzyme involved in the tricarboxylic acid cycle. This enzyme catalyzes the oxidative decarboxylation of isocitrate acid to aketoglutaric (a-KG) in an NADP^+^-dependent manner by using divalent magnesium ion (Jiao et al., 2016), which is related to the progression of various tumors, including acute myeloid leukemia, gliomas, and other solid tumors (Yan and Reitman, 2010; Yen et al., 2016).

Somatic mutations of IDH1 have been frequently identified in many types of cancer, including approximately 80% of grade II-III gliomas, nearly 45% of secondary glioblastoma multiforme (GBM), and 33%-50% of adult primitive neuroectodermal tumors (Dang et al., 2009; Wang et al., 2013). IDH1 mutations have also been discovered in other cancers, such as colorectal cancer (Xu et al., 2011), acute myeloid leukemia (Parsons et al., 2008), and prostate cancer (Hartmann et al., 2009). Key amino acid residue Arg132 is the most common mutation in IDH1, which is located in the catalytic pocket (Dang et al., 2009). Specific mutations belong to heterozygous missense mutations and lead to a new form of IDH1 catalytic activity, which convert α-KG into an oncometabolite D2-hydroxyglutarate (Dang et al., 2009). The oncometabolite (D2-HG) is associated with tumorigenesis, which impairs hematopoietic differentiation and promotes leukemia by inducing the hypermethylation of histone and chromatin and preventing cell differentiation (Figueroa et al., 2010; Xu et al., 2011). Due to the IDH1 mutation, high levels of D2-HG are created that promote the occurrence and development of cancers, such as gliomas (Parsons et al., 2008) and acute myeloid leukemia (Mardis et al., 2009). Therefore, although the contribution of IDH1 mutants to carcinogenic properties has yet to be elucidated, IDH1 mutants have become therapeutic targets for cancer, especially AML.

**Figure 1:**
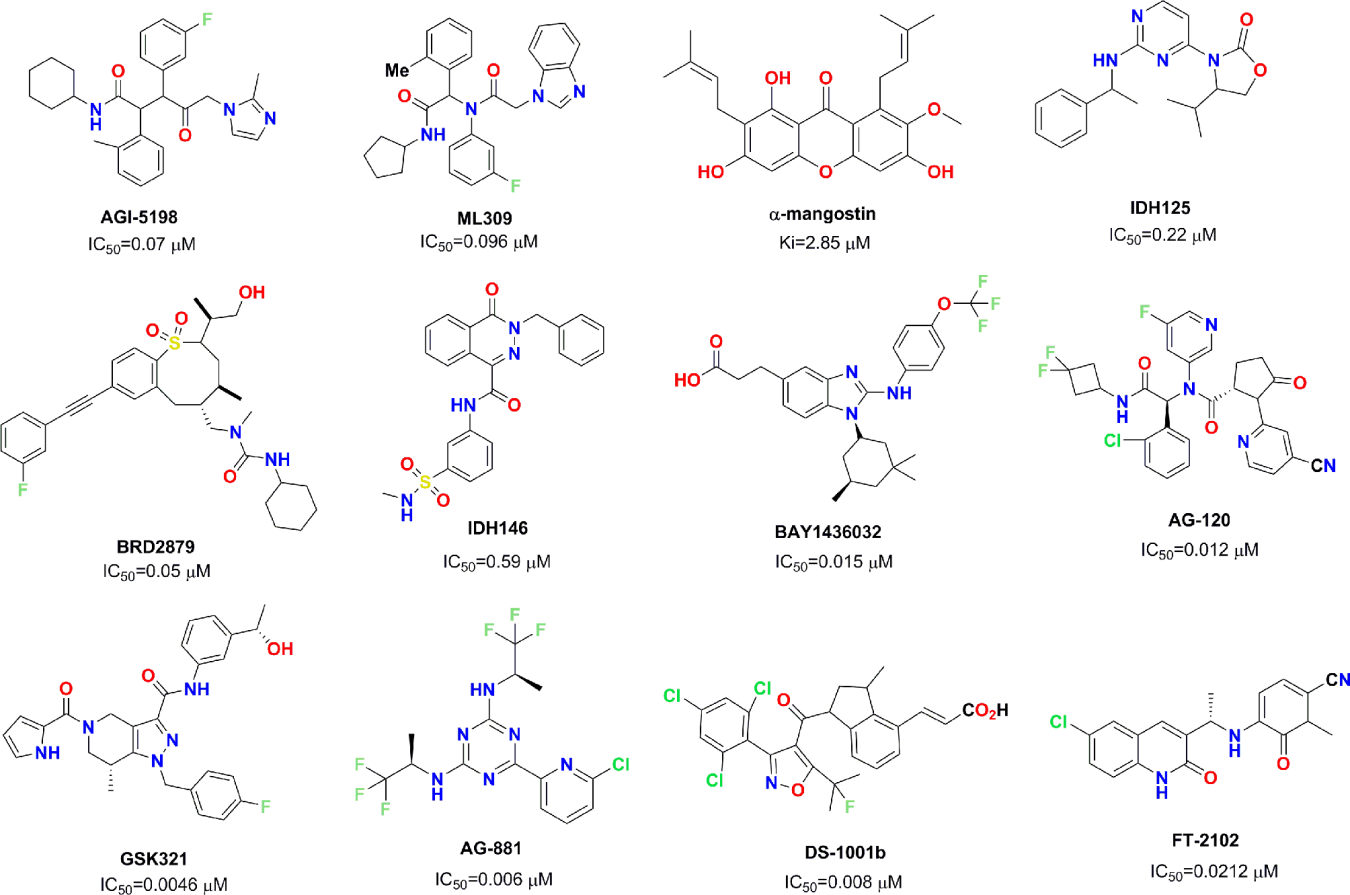
Chemical skeleton of nine representative IDH1^R132H^ inhibitors.

Mutant IDH1 has become a very attractive therapeutic target in the field of antitumor drug discovery, and several pharmaceutical companies have attempted to develop novel small molecule inhibitors against mutant IDH1. So far, several small molecule inhibitors targeting mutant IDH1 enzymes have been developed (see **Figure 1**) (Rohle et al., 2013; Davis et al., 2014; Deng et al., 2015; Kim et al., 2015; Okoye-Okafor et al., 2015; Law et al., 2016; Chaturvedi et al., 2017; Xie et al., 2017; Popovici-Muller et al., 2018; Nakagawa et al., 2019; Caravella et al., 2020; Konteatis et al., 2020). Some of these have been studied in various preclinical models, and some are currently being evaluated in phase I/II clinical studies for different tumor pathologies with IDH1 enzyme mutations. AG-120 as the only mutant IDH1 inhibitor in clinic approved by the FDA that has shown encouraging clinical benefits with a total overall response rate of 42% for advanced hematological malignancies (Foran et al., 2019). In light of these encouraging finding, we employed docking-based virtual screening to identify active hits with novel skeleton for targeting mutant IDH1.

Structure-based virtual screening is now widely used in early-stage drug discovery (Sheisi et al., 2019), and has been applied to the discovery of IDH1 inhibitors. To date, there have been several attempts to identify potential IDH1 inhibitors by using structure-based virtual screening in terms of the reported crystal structures of the IDH1 complex (Zou et al., 2016; Zheng et al., 2017; Zou et al., 2018). In 2016, by using a docking-based virtual screening strategy (PDB: 4UMX), Zou et al. identified a series of IDH1 inhibitor FX-03 with IC_50_ values of 55.50 μM and 68.38 μM in HEK-293T cells transfected with IDH1 R132H and IDH1 R132C, respectively (Zou et al., 2016). Importantly, FX-03 exhibited significant selectivity between the IDH1^WT^ and IDH1^R132H^ mutants. In 2017, Zheng et al. discovered a natural product, clomifene, as an effective inhibitor against the IDH1^R132H^ mutant with a K_d_ value of 18.45 μM by using docking-based virtual screening (PDB: 4UMX) (Zheng et al., 2017). They also proved that clomifene selectively inhibits mutant IDH1 activities *in vitro* and *in vivo* models. It should be noted that, although these studies have identified several IDH1^R132H^ inhibitors, they used the same IDH1^R132H^ crystal structure in structure-based virtual screening. Considering the difference in binding mode after the binding of various ligands, comparing the virtual screening capabilities of different IDH1^R132H^ crystal structures based on docking-based virtual screening appears to a more reasonable strategy to discover potential IDH1^R132H^ inhibitors.

**Figure 2:**
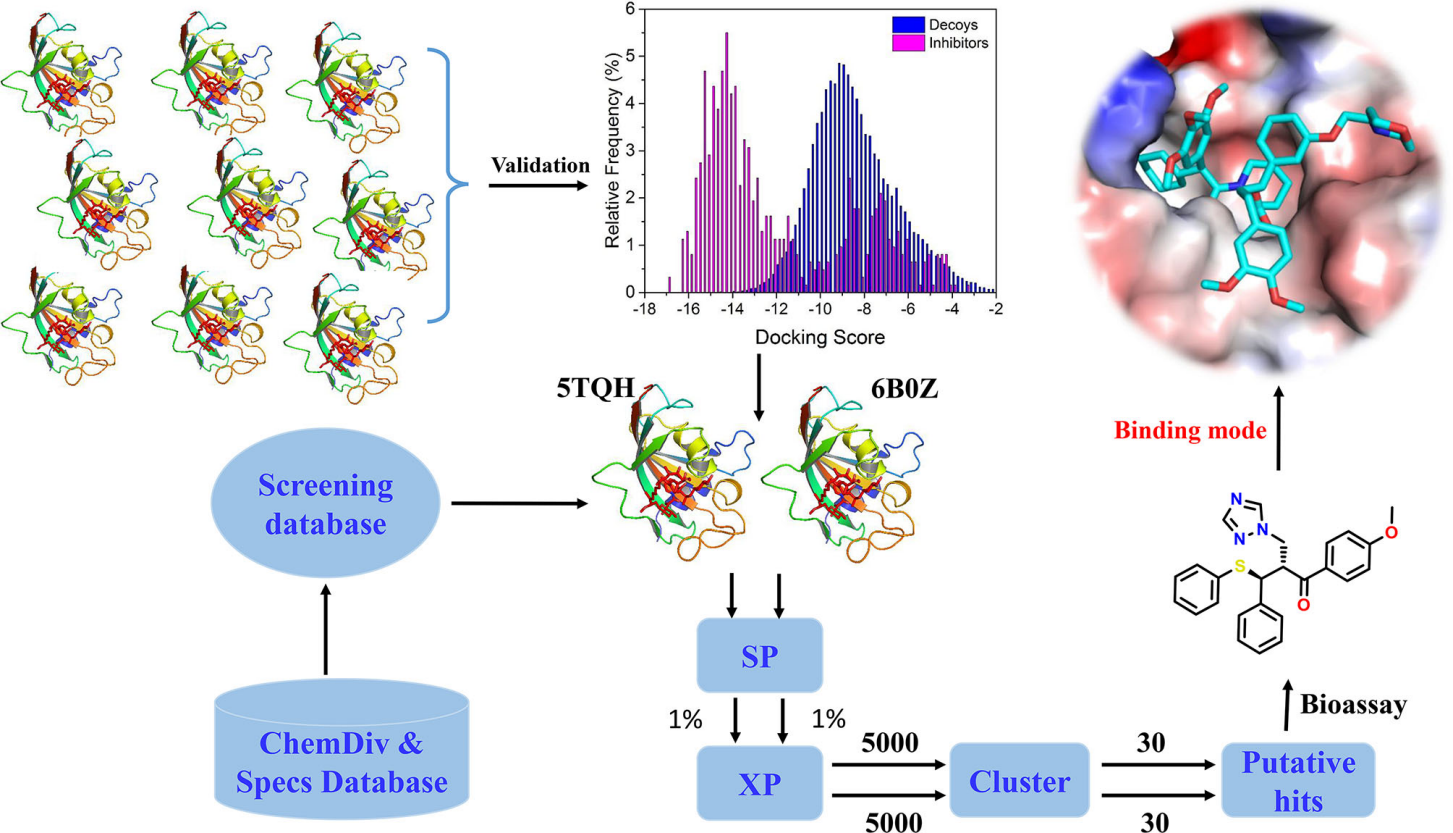
The workflow of docking-based virtual screening and bioassay for IDH1^R132H^ inhibitor.

In the present study, the performance of docking-based virtual screening for nine crystal structures of IDH1^R132H^ were compared through a combination of docking power and screening power. Two best performing IDH1^R132H^ complexes were employed to identify potential IDH1^R132H^ inhibitors with diverse structures from ChemDiv (http://www.chemdiv.com) and Specs (http://www.specs.net) databases. Followed by further examination and verification, a series of compounds with novel skeleton were addressed and could be used as IDH1^R132H^ inhibitors. The overall workflow was shown in **Figure 2**.

## Materials and Methods

### Preparation of Crystal Structures and Data Sets

The crystal structures of the IDH1^R132H^ in complex with an inhibitor were downloaded from the PDB database (http://www.rcsb.org), including 4UMX, 5L57, 5L58, 5LGE, 5SUN, 5SVF, 5TQH, 6ADG, and 6B0Z. For each complex, the *Protein Preparation Wizard* module in Schrödinger 2015 (Schrödinger, LLC, New York, NY, 2015) was applied to add hydrogen and missing side chains, remove all water molecules, assign protonation states and partial charges through OPLS2005 force field (Jorgensen et al., 1996), and minimize all heavy atoms until the root-mean-square deviation (RMSD) was reached ≤0.3 Å.

To evaluate the virtual screening capability of different crystal structures, 423 actives were directly extracted from the PubChem database and served as a validation data set (https://pubchem.ncbi.nlm.nih.gov/bioassay/1344832#section=Top), and their decoys, generated by DUD•E (Mysinger et al., 2012), were considered as a decoy data set. In total, 23,900 decoys were generated.

### Evaluate the Performance of Each Structure

In order to discover the favorable crystal structure for virtual screening, the docking performance of each IDH1^R132H^ structure was systematically evaluated. All actives and decoys were preprepared using the LigPrep (LigPrep, Schrödinger, LLC, New York, NY, 2015) module in the Schrödinger package. The possible ionized states of each compound were calculated by using Epik (Shelley et al., 2007) at pH= 7.0 ± 2.0. The chirality of the IDH1^R132H^ inhibitors with 3D structures were preserved, while the chirality of the decoys was determined from 3D structures based on the different combinations. The stereoisomers for each ligand generated, at most, 32, and the other parameters were set to default values. Subsequently, a grid box of each complex was generated by using the Receptor Grid Generation module of Schrödinger software, which was centered at the native ligand of the complex and defined as a similar size to the native ligand space. Finally, all chemicals in the validation set and decoy set were docked into the binding site of each IDH1^R132H^ complex in turn and evaluated by using the standard precision (SP) and extra precision (XP) scoring function of Glide. In order to choose the best crystal structure of IDH1^R132H^ for virtual screening, the enrichment factor (EF) (Halgren et al., 2004) was used to evaluate the virtual screening capability of each model, which was defined as the following Equation:

$$EF\frac{\frac{Hits_{set}}{n}}{\frac{Hits_{all}}{N}}$$

where Hits_set_ is the number of actives in the selected subset n of the ranked database and Hits_all_ is the total number of actives in the database. The model with the highest EF value was reserved and used to screen potential IDH1^R132H^ inhibitors.

### Docking-Based Virtual Screening

All compounds in the ChemDiv and Specs database were first preprocessed according to the method of the above step, and then screened by docking-based virtual screening against two IDH1^R132H^ crystal structures (PDB ID: 5TQH and 6B0Z). After the possible ionized states and tautomer were calculated at pH=7.0 ± 2.0 by using Epik module, the chirality of each compound was determined from 3D structures; the stereoisomer for each ligand generated, at most, 32. The final virtual screening library was generated to include approximately 2 million compounds, and then initially filtered by Lipinski’s Rule, removing ligands with reactive functional groups. Finally, docking-based virtual screening was employed by use of the high throughput virtual screening (HTVS) scoring function, SP scoring function, and XP scoring function of Glide in sequence. In the screening process at each step, 10% of the best compounds were reserved for further analysis.

### Clustering Analysis

The reserved compounds after docking-based virtual screening were structurally clustered into 30 clusters by using K-means clustering on the MACCS structural keys in Canvas (Canvas, Schrödinger, LLC, New York, NY, 2015), and the compound in each cluster with the lowest docking score was selected. In the end, 60 chemicals were eventually submitted to purchase from Topscience Co., Ltd (https://www.tsbiochem.com).

### Enzymatic Assay

The primary assay was carried out in 10 μL of base buffer (10 mM MgCl2, 20 mM Tris pH7.5, 150 mM NaCl, 0.05% (w/v) bovine serum albumin) containing 2.5 μL of the test compound, 5 μL of an enzyme solution (0.3 ng/μL mutant IDH1^R132H^), and 2.5 μL of a substrate solution (4 mM α-KG, 16 μM NADPH). This assay added into a 384-well blank plate and then incubated at room temperature for 60 min. The secondary assay, with 5 μL of base buffer containing 15 μM resazurin and 0.01 unit diaphorase, was added to the entire plate and incubated at room temperature for 10 min. Florescence was read on a SYNERGY^H1^ microplate reader (BioTek) at Ex 540 Em 590. Curve fitting for dose response IC_50_ was done using GraphPad Prism.

## Results and Discussion

### Performance of the Nine IDH1^R132H^ Complex

As a significant indicator of the docking reliability, docking power was used to reveal the binding pose of the experiment between small molecules and proteins, which was mainly evaluated after redocking with the RMSD value of the docking pose and native pose of the small molecule in the IDH1^R132H^ complex. For each IDH1^R132H^ complex, after the native inhibitor was separated from the corresponding complex and preprepared, it was redocked into the original binding site. The RMSD value between the native conformation of the inhibitor and the docked pose for each crystal structure was respectively computed, and RMSD ≤ 2.0 Å served as the evaluation standard to verify the docking reliability. It can be seen from **Table 1** that Glide docking could identify the near-native pose of most inhibitors in IDH1^R132H^ crystal structures by using the XP or SP scoring function in Glide.

**Table 1:** The summary of the docking power of molecular docking in glide for nine IDH1^R132H^ crystal structures.

PDB	Ligand	SP		XP	
		Docking score	RMSD	Docking score	RMSD
4UMX	VVS	-7.13	2.16	-7.04	2.10
5L57	6N3	-8.50	2.32	-9.23	2.56
5L58	6MX	-9.38	1.56	-9.97	1.59
5LGE	6VN	-6.85	0.90	-7.18	1.79
5SUN	70Q	-10.31	5.12	-10.57	5.11
5SVF	70P	-9.74	1.06	-12.94	0.44
5TQH	7J2	-12.75	0.95	-16.12	0.60
6ADG	9UO	-5.74	0.66	-5.61	1.39
6B0Z	C81	-12.53	0.45	-15.52	1.07

**Figure 3:**
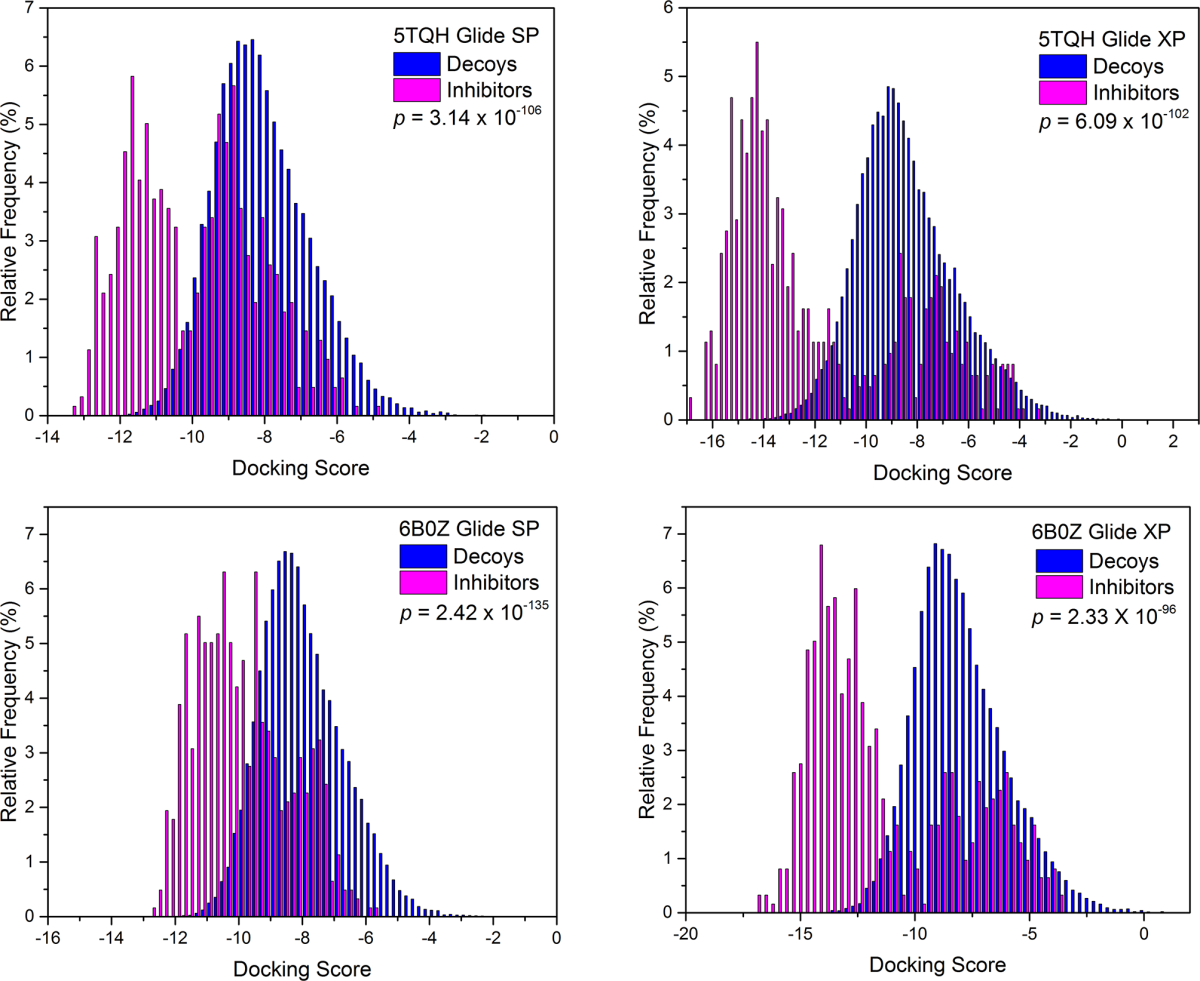
The distributions of Glide docking scores of validation sets for two IDH1^R132H^ crystal structures with the best screening power.

Next, screening power of glide docking was used to identify the reported inhibitors from the decoys in nine IDH1^R132H^ complexes, and these were compared and calculated. In contrast with the docking power of glide docking, the screening power of each crystal structure is a more important index for the docking-based virtual screening process. Herein, we performed student’s t test to evaluate the significant difference between the means of the two distributions of the Glide XP or SP scores for the known actives and decoys. It can be seen from **Table 2** that molecular docking of Glide can efficiently discriminate the IDH1^R132H^ inhibitors from the decoys in nine complexes of IDH1^R132H^ based on the relatively low *p* value. The area under the receiver operating characteristic curve (AUC-ROC), EF, and Robust Initial Enhancement (RIE) were also employed to comprehensively evaluate the screening capabilities of each crystal structure. As shown in **Figure 3**, the best screening power (*p* value = 2.42x10^-135^, AUC-ROC=0.96, and RIE= 12.88) was acquired by using SP scoring function and 6B0Z was reserved as the screening template. However, 5TQH exhibited the best screening power (*p* value = 6.09x10^-102^, AUC-ROC=0.96, and RIE= 15.76) in XP scoring function, which was also retained as a screening complex. Our results suggest that that it is necessary to compare the performance of different complexes in the process of virtual screening.

**Table 2:** The summary of the screening power of molecular docking in glide for nine IDH1^R132H^ crystal structures for validation set.

PDB ID	SP Precision								XP Precision							
	*p* value	AUC-ROC	RIE	EF^1%^	EF^2%^	EF^5%^	EF^10%^	EF^20%^	*p* value	AUC-ROC	RIE	EF^1%^	EF^2%^	EF^5%^	EF^10%^	EF^20%^
4UMX	3.51x10^-100^	0.53	0.37	0.47	0.35	0.33	0.33	0.43	1.16x10^-45^	0.88	7.5	13	14	9.3	6.2	3.9
5L57	1.84x10^-6^	0.7	1.06	0.95	0.95	0.95	0.97	1.4	6.15x10^-8^	0.76	2.15	0.71	1.3	2	2.5	2.4
5L58	5.20x10^-21^	0.76	2.21	0.94	2.4	2.6	1.9	2	1.81x10^-15^	0.82	4.52	6.8	7.3	5.3	4	2.9
5LGE	1.25x10^-3^	0.72	1.31	0.71	0.83	1	1.4	1.8	1.08x10^-10^	0.77	2.61	3.8	3.1	2.5	2.6	2.4
5SUM	5.67x10^-17^	0.77	1.86	0.71	1.4	1.6	1.9	2.2	2.50x10^-16^	0.84	3.48	0.71	1.5	3.5	4.2	3.6
5SVF	7.33x10^-87^	0.91	10.38	31	24	12	7.2	4	8.91x10^-87^	0.95	14.95	62	39	17	9	4.6
5TQH	3.14x10^-106^	0.92	11.82	50	30	13	7.1	4.1	6.09x10^-102^	0.96	15.76	74	43	18	9	4.6
6ADG	1.12x10^-50^	0.9	9.4	31	22	11	6.4	4	1.60x10^-44^	0.9	9.56	29	21	11	6.7	4
6B0Z	2.42x10^-135^	0.96	12.88	46	30	15	8.3	4.7	2.33x10^-96^	0.96	15.43	70	41	18	8.9	4.5

### Structure-Based Virtual Screening

The overall workflow of structure-based virtual screening was shown in **Figure 2**. The Specs and ChemDiv database, which consisted of more than 2,100,000 compounds, have been used for virtual screening of small molecule databases. Firstly, Lipinski’s rules of five was employed to filter compounds that did not meet the criteria, and then these compounds containing PAINS substructures were also removed. A total of 1.46 million compounds were retained. According to the MACCS structural fingerprint, residual chemicals were structurally clustered in 30 clusters *via* K-means clustering in Canvas, and the chemical with the lowest docking score in each cluster was retained was retained (see **Table 3**). Finally, a total of 57 chemicals were purchased and tested based on the docking-based virtual screening on two IDH1 complex (5TQH and 6B0Z).

**Table 3:** The molecular weight and docking score for putative hits.

ID	MW	Docking score (kcal/mol)
6470-0047	473.524	-15.53
G420-0655	460.55	-15.30
C798-1008	456.561	-15.25
E894-1127	469.539	-15.21
V004-0504	488.618	-15.17
L710-2843	447.49	-14.94
G389-1098	464.495	-14.72
S383-0082	412.438	-14.47
D103-1045	473.545	-14.45
C647-0812	484.551	-14.43
D491-0852	435.524	-14.40
V015-1671	491.426	-14.39
S733-2152	475.51	-14.37
V016-3750	453.515	-14.25
L710-0317	419.479	-14.20
L970-0181	487.529	-14.12
5782-4343	407.465	-14.11
V020-6264	478.931	-14.10
F019-2828	374.485	-14.09
M506-0358	404.44	-14.00
G741-1212	466.898	-13.96
S631-0764	421.513	-13.96
V022-0932	414.503	-13.95
D217-0418	416.454	-13.88
D336-7545	441.544	-13.78
V020-8255	472.54	-13.77
AQ-714/41674992	429.536	-13.62
M136-0372	474.949	-13.47
K781-3358	464.338	-13.39
3601-0061	426.452	-13.01
AQ-149/42126332	488.536	-15.34
V010-1281	478.555	-15.23
E867-1033	462.522	-15.14
V028-6550	490.53	-15.13
G800-0501	488.53	-15.09
C798-1007	476.979	-14.92
E894-1218	469.539	-14.81
V013-4787	435.524	-14.67
V025-9252	467.951	-14.51
AK-778/43465022	494.341	-14.45
V025-7538	496.485	-14.38
V003-2610	458.488	-14.18
K297-1090	474.576	-14.17
M136-0633	474.949	-14.11
8019-1512	410.398	-14.09
V001-8209	458.909	-14.05
F521-0664	486.526	-13.83
L487-0168	459.476	-13.78
G798-0506	434.534	-13.74
C647-0805	454.524	-13.68
J108-0614	432.478	-13.63
D349-0203	442.473	-13.62
L806-0255	457.842	-13.53
F815-0210	440.494	-13.53
C769-0129	438.54	-13.46
V020-4317	465.351	-13.40
G568-0082	454.973	-13.32
E867-0977	452.957	-13.27
V005-6943	477.534	-13.08

### IDH1^R132H^ Enzymatic Assay

**Figure 4:**
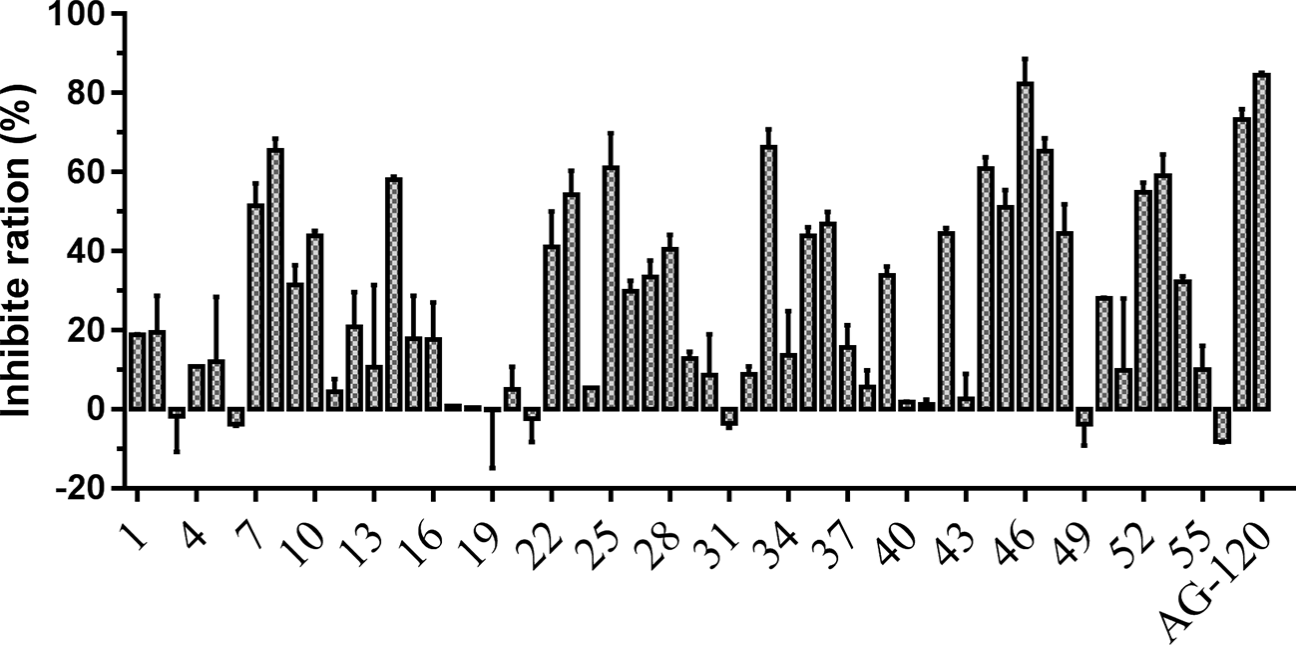
Inhibitory activity of the 57 candidates at 50μM. The bars indicate the inhibitory activity of chemicals targeting IDH1^R132H^. AG-120 at 100 nM was used as the positive control.

**Figure 5:**
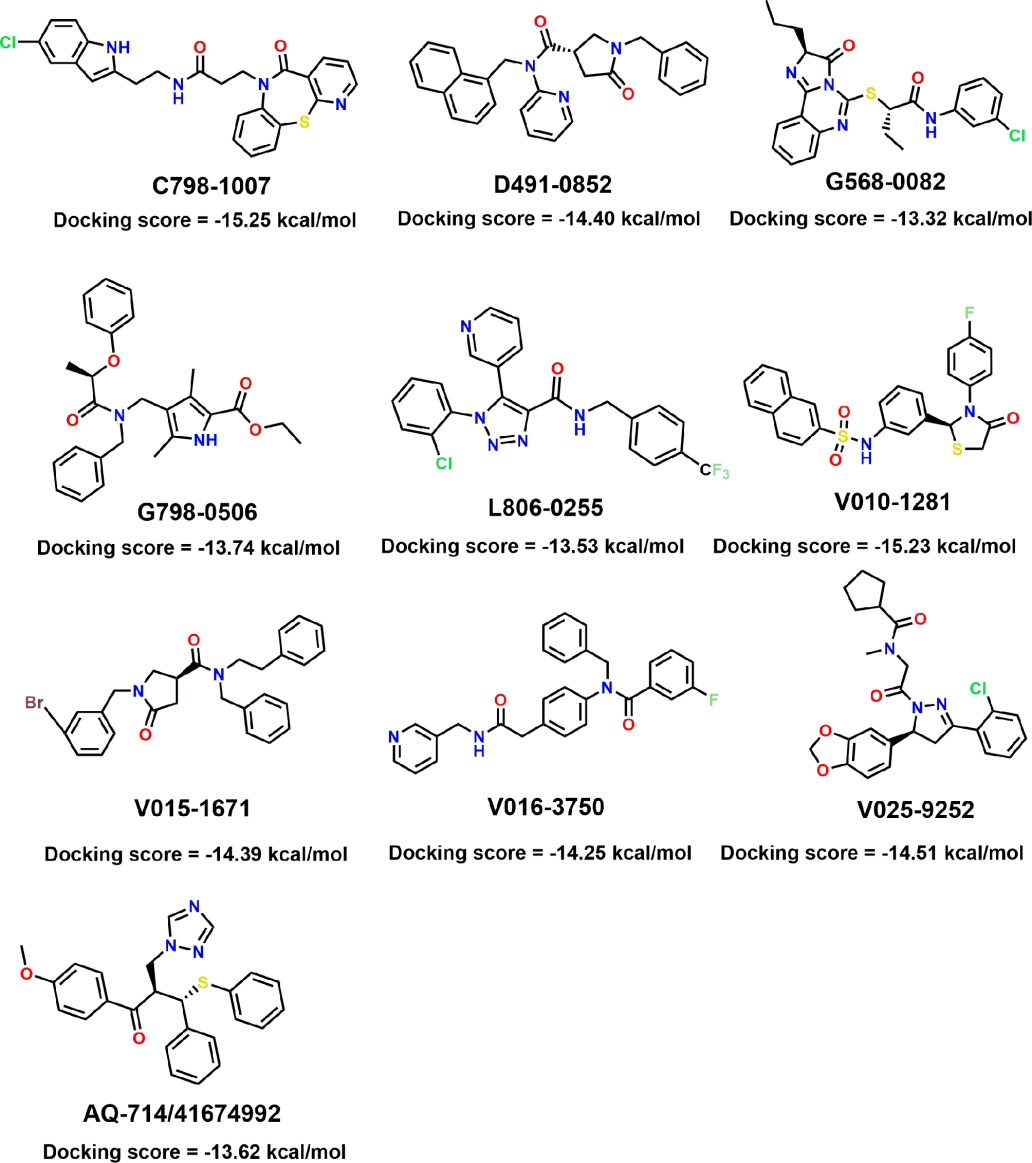
Molecular structures of the 10 selected IDH1^R132H^ inhibitors by using based-docking virtual screening.

**Figure 6:**
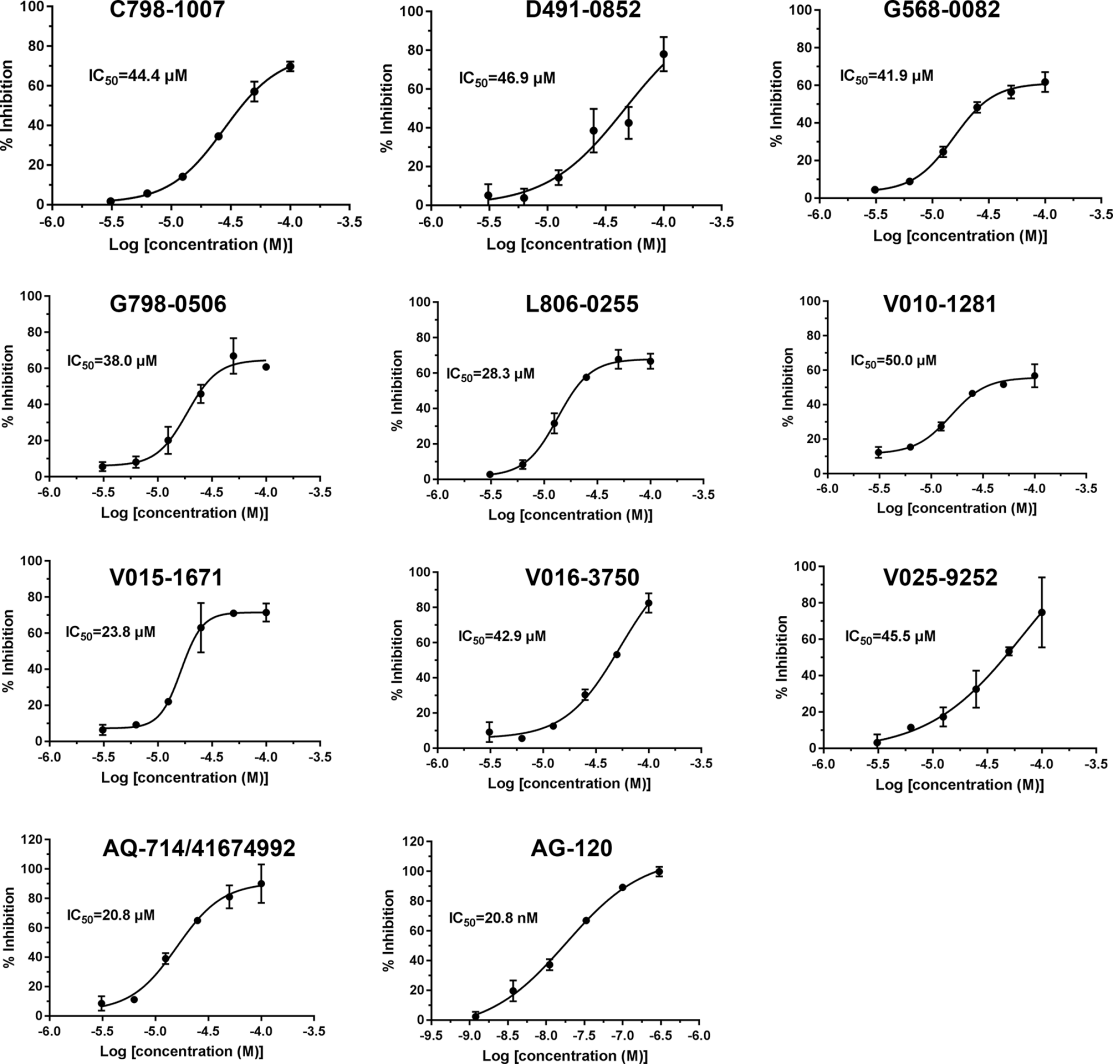
IDH1^R132H^ enzymatic inhibition of 10 identified small molecule inhibitors.

To verify the inhibitory activity of screening compounds targeting IDH1^R132H^, enzyme activity assay was performed. As shown in **Figure 4**, we found that 12 compounds (7, 8, 14, 23, 25, 33, 44, 46, 47, 52, 53, and 57) exhibited over 50% inhibition at 50 μM. These 12 ligands were submitted to determine the IC_50_. It can be seen from **Table 4** that 10 of them show IC_50_ ≤ 50 μM. Molecular structures of the 10 selected compounds of IDH1^R132H^ are exhibited in **Figure 5**. The enzymatic curves and docking score for these 10 compounds against IDH1^R132H^ are depicted in **Figure 6**. Tanimoto coefficient (Tc) (Willett and Winterman, 1986; Willett et al., 1986), in terms of the ECFP4 fingerprint, was calculated to compare the structural similarity between 10 compounds and reported inhibitors. As shown in **Figure S1**, we can find that putative hits have low similarity with reported inhibitors (Tc < 0.2). Therefore, these compounds are structurally new and have the potential to be promising leads for further optimizations.

**Table 4:** The summary of the inhibition ratio of 10 candidate compounds by using virtual screening.

No.	Database	PDB	Inhibition ratio (%) 50 (μM)	Enzymatic
				IC_50_ (μM)
C798-1007	ChemDiv	6B0Z	65.45±2.15	44.4±1.3
D491-0852	ChemDiv	5TQH	58.00±0.60	46.9±6.0
G568-0082	ChemDiv	6B0Z	54.30±4.30	41.9±8.0
G798-0506	ChemDiv	6B0Z	61.15±6.15	38.0±2.0
L806-0255	ChemDiv	6B0Z	66.25±3.25	28.3±2.5
V010-1281	ChemDiv	6B0Z	60.85±2.05	50.0±6.4
V015-1671	ChemDiv	5TQH	65.30±2.30	23.8±1.8
V016-3750	ChemDiv	5TQH	54.90±1.70	42.9±2.8
V025-9252	ChemDiv	6B0Z	59.15±3.75	45.5±3.1
AQ-714/41674992	Specs	5TQH	73.30±1.90	20.8±4.2
AG-120	–	–	84.40±0.50 (nM)	16.7±1.7 (nM)

### Binding Mode Prediction

**Figure 7:**
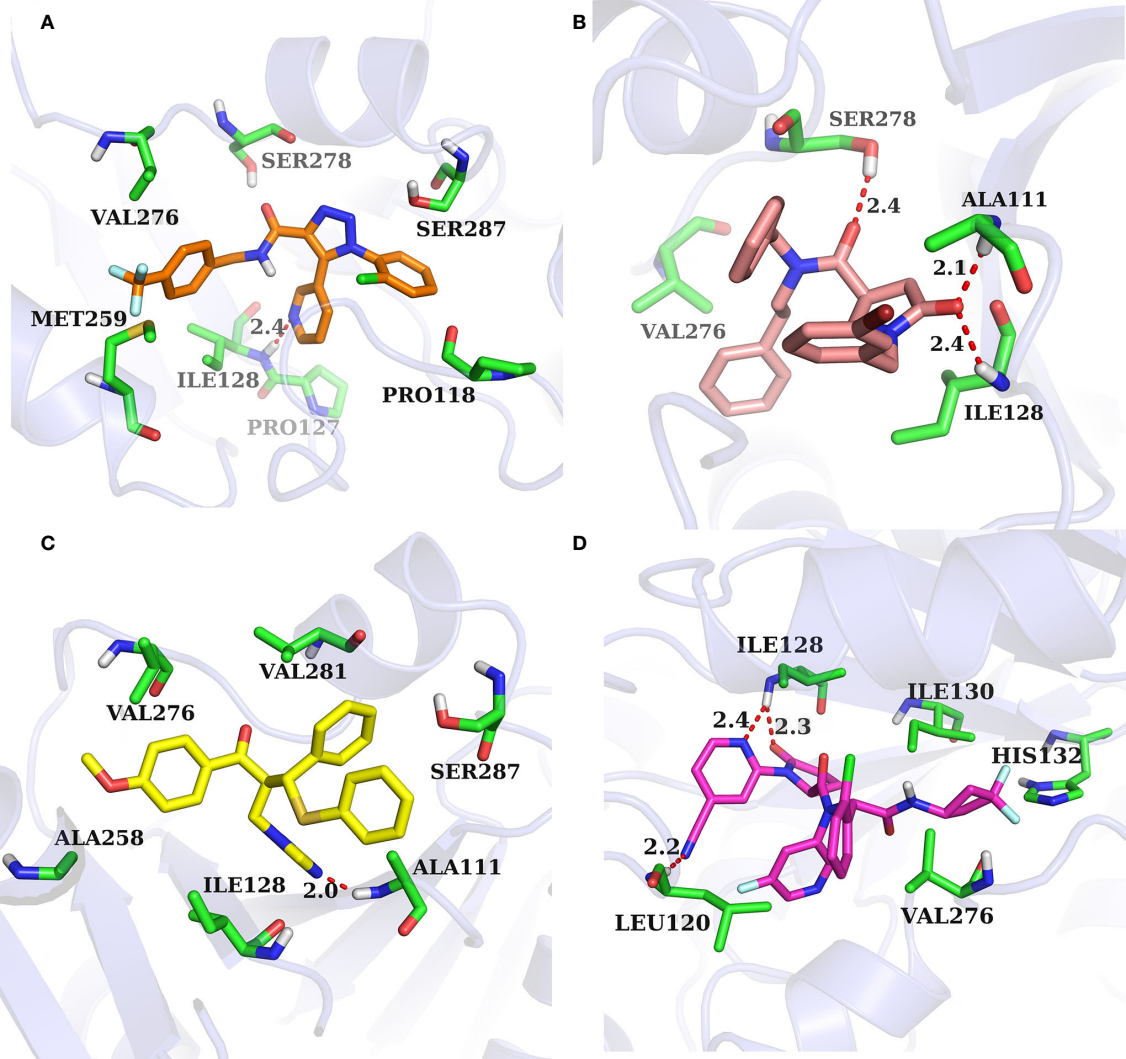
Binding mode of identified IDH1^R132H^ inhibitor and AG-120. **(A)** L806-0255. **(B)** V015-1671. **(C)** AQ-714/41674992. **(D)**AG-120.

The binding pocket of IDH1 lies on the dimer interface and most of the reported compounds have been shown to bind to this allosteric site. In order to gain insight into the structural basis of the identified IDH1^R132H^ inhibitor, the binding mode of the three compounds was compared with AG-120. As shown in **Figure 7**, three molecules that could be docked into the binding pocket consisted of hydrophobic residues in a similar manner to AG-120, and formed intermolecular hydrogen bonds with key residues, which stabilized the complex. L806-0255 and V015-1671 form a key hydrogen bond with ILE128, which is consistent with AG-120. In addition, V015-1671 and AQ-714/41674992 also form a key hydrogen bond with ALA111. Moreover, the hydrophobic contacts formed between surrounded residues, such as VAL276, SER278, SER287, ILE128, PRO118, and compounds also contribute to enhanced binding of the small molecule inhibitor to IDH1 ^R132H^. Therefore, the above results suggested that L806-0255, V015-1671, and AQ-714/41674992 could bind to IDH1^R132H^.

## Conclusions

In the present work, we first verified the performance of IDH1^R132H^ by using glide-docking-based virtual screening and discovered two crystal structures with the most credible screening ability. Based on the best performing crystal structure, docking-based virtual screening was performed to identify new IDH1^R132H^ inhibitors. A total of 57 potential hits were purchased and their activity against IDH1^R132H^ was addressed, and 10 of them exhibited anti-IDH1^R132H^ activity.

## Data Availability Statement

All datasets presented in this study are included in the article/**Supplementary Material**.

## Author Contributions

HG, XY, and EL conceived this study and revised the manuscript. YW, ST, HL, RJ, and XL carried out the experiments and analyzed the experimental data. YW and XY wrote the manuscript. All authors reviewed the manuscript. All authors contributed to the article and approved the submitted version.

## Funding

This work was supported by the National Natural Science Foundation of China (82003653), the Shaanxi University of Chinese Medicine (Project No. 2020XG01), the Subject Innovation Team of Shaanxi University of Chinese Medicine (Project No. 2019-PY02), and Macau Science and Technology Development Fund (Project Nos. 005/2014/AMJ, 082/2015/A3, 046/2016/A2 and 086/2015/A3).

## Conflict of Interest

The authors declare that the research was conducted in the absence of any commercial or financial relationships that could be construed as a potential conflict of interest.

## Supplementary Material

Supplementary Figure 1 The Tanimoto coefficient matrix based on ECFP4 fingerprint of identified IDH1^R132H^ inhibitor *vs* reported IDH1^R132H^ inhibitor.
